# Room-temperature synthesis of three-dimensional porous ZnO@CuNi hybrid magnetic layers with photoluminescent and photocatalytic properties

**DOI:** 10.1080/14686996.2016.1165583

**Published:** 2016-04-14

**Authors:** Miguel Guerrero, Jin Zhang, Ainhoa Altube, Eva García-Lecina, Mònica Roldan, Maria Dolors Baró, Eva Pellicer, Jordi Sort

**Affiliations:** ^a^ Departament de Física, Facultat de Ciències, Universitat Autònoma de Barcelona, E-08193Bellaterra, Spain; ^b^ Surfaces Division, IK4-CIDETEC, Paseo Miramón, 196, E-20009San Sebastián, Spain; ^c^ Servei de Microscòpia, Universitat Autònoma de Barcelona, E-08193Bellaterra, Spain; ^d^ Institució Catalana de Recerca i Estudis Avançats (ICREA) and Departament de Física, Universitat Autònoma de Barcelona, E-08193Bellaterra, Spain

**Keywords:** Porous nanocomposite, CuNi alloy, ZnO nanoparticles, ferromagnetism, photoluminescence, 40 Optical, magnetic and electronic device materials, 102 Porous / Nanoporous / Nanostructured materials, 100 Materials, 103 Composites, 205 Catalyst / Photocatalyst / Photosynthesis, 200 Applications, 203 Magnetics / Spintronics / Superconductors

## Abstract

A facile synthetic approach to prepare porous ZnO@CuNi hybrid films is presented. Initially, magnetic CuNi porous layers (consisting of phase separated CuNi alloys) are successfully grown by electrodeposition at different current densities using H_2_ bubbles as a dynamic template to generate the porosity. The porous CuNi alloys serve as parent scaffolds to be subsequently filled with a solution containing ZnO nanoparticles previously synthesized by sol-gel. The dispersed nanoparticles are deposited dropwise onto the CuNi frameworks and the solvent is left to evaporate while the nanoparticles impregnate the interior of the pores, rendering ZnO-coated CuNi 3D porous structures. No thermal annealing is required to obtain the porous films. The synthesized hybrid porous layers exhibit an interesting combination of tunable ferromagnetic and photoluminescent properties. In addition, the aqueous photocatalytic activity of the composite is studied under UV−visible light irradiation for the degradation of Rhodamine B. The proposed method represents a fast and inexpensive approach towards the implementation of devices based on metal-semiconductor porous systems, avoiding the use of post-synthesis heat treatment steps which could cause deleterious oxidation of the metallic counterpart, as well as collapse of the porous structure and loss of the ferromagnetic properties.

## Graphical abstract

**Figure:**
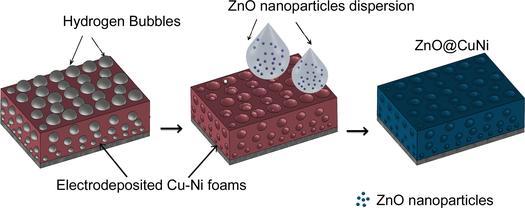

## 1. Introduction

The advances in the synthetic pathways to produce porous semiconducting materials (PSM) with controllable pore size and composition have boosted a wealth of applications in a number of different fields such as solar fuel production,[1] gas-sensing,[2,3] or environmental healing,[4,5] where materials with a high surface area are essential. Different PSM architectures including particles, rods, wires and films (the latter with porosity ranging from the macro- to the nanoscale) have been engineered in recent years.[6] Depending on the end application, PSMs have been manufactured either alone or in combination with other materials.

In general, the ability to use three-dimensional (3D) porous scaffolds as parent templates to be filled or coated with second phase materials (e.g. oxide semiconductors) has been shown to trigger new functionalities stemming from the synergy created between the host and the guest (or the matrix and the filler) components.[7] For example, in the case of metal-oxide ceramic composites, one can take advantage of the large hardness of the ceramic counterpart and the high ductility of the metal to design microstructures with optimized mechanical performance.[8,9] Magnetic exchange-interactions in certain metal-oxide nanocomposites have also triggered interesting applications in the field of nanomagnetism, as a means to enhance the coercivity of the resulting material.[10]

One of the main challenges to produce hybrid composite materials using polymeric or metallic porous scaffolds as templates, particularly when the second phase is intended to be an oxide, is that a high-temperature heat-treatment, often in the presence of an O_2_-containing atmosphere, is required.[11] Such treatment can cause carburization of the polymer or oxidation of the parent metallic template, and can eventually provoke the collapse of the porous network. These issues need to be tackled when growing a metal oxide as the second phase by atomic layer deposition or colloidal templating (which typically involves subsequent heat-treatment in air).[11] Thus, new methodologies to fill or coat porous templates with suitable second-phase materials at temperatures close to room temperature are required.

Zinc oxide (ZnO), one of the most important n-type II-VI semiconductor materials with a wide band gap of 3.2 eV, possesses unique electrical and optical properties including good transparency, high electron mobility, and strong room-temperature luminescence.[12] Therefore, ZnO has recently attracted enormous attention due to its potential applications in integrated optoelectronics and laser technology[13] or photocatalysis.[14,15] Additionally, the piezo/pyroelectric properties of ZnO make it a smart candidate for sensors and other types of micro/nano-electromechanical systems (MEMS/NEMS).[16, 17] There are several synthetic techniques for the growth of ZnO-based materials. These include vapor deposition,[18] hydrothermal synthesis,[19] the sol-gel method,[20] or mechanochemical approaches.[21] Depending on the deposition process conditions, different structural, electrical and optical properties are obtained. Pulsed laser deposition,[22] chemical vapor deposition[23] or spray pyrolysis[24] can be used to prepare dense ZnO immobilized films. The synthesis of porous ZnO films is typically more challenging. Dip-coating methods of sol-gel precursors are receiving attention since they enable the low-cost deposition of ZnO films, eventually in porous form.[25, 26] Other approaches to synthesize porous metal oxide films on a substrate include spin-coating of sol-gel[27], template-assisted electrostatic spray deposition[28] and hydrothermal methods.[29] Both dip-coating and spin-coating of sol-gel mixtures show significant limitations when they are faced with large substrates and/or substrates with micrometer-sized features. Moreover, film thickness typically achieved in a single coating step is in the range of about 100−400 nm depending on coating conditions, whereas a film thicker than 1 μm can be obtained only by repeated coating/calcination cycles.[28] For the precise control of the film thickness, pore size and porosity degree, the use of pre-fabricated polymeric or metallic porous templates is often preferred. Nevertheless, in most cases, one or more post-synthesis heat treatment steps at temperatures ranging from 250°C to 900°C are necessary to convert the solution precursors into ZnO.[30, 31] As aforementioned, this can be a serious shortcoming when the porous scaffold is not intended to be eliminated but to remain unaffected (and hence keep the initial features like the composition, overall porosity and physical properties) after the heat treatment.

In this work, a novel approach to coat the interior of the pores of a CuNi 3D porous film scaffold with a ZnO shell at room temperature is presented. CuNi films are obtained by hydrogen-bubble assisted electrodeposition, which has proven to be a convenient technique to produce networked macroporous films of some compositions.[32] The Cu/Ni ratio is adjusted in order to deliver room-temperature ferromagnetic properties, which are not typically available in single-phase semiconductor materials. Thus, the obtained hybrid porous composites maintain the magnetic properties of the CuNi foams while exhibiting new functionalities (e.g. photoluminescence, photocatalytic activity) stemming from the presence of the ZnO semiconducting second phase. In particular, the aqueous photocatalytic activity of this composite system under UV-visible light irradiation for the degradation of Rhodamine B (RhB) is demonstrated. The multifunctional character of this composite material is possible because (i) the macro-porosity of the initial CuNi alloy foam is preserved and (ii) oxidation of the CuNi parent template is also avoided because of the low temperature used in all the synthetic steps.

## 2. Experimental section

### 2.1. Synthesis of the CuNi metallic foams

**Figure 1.**
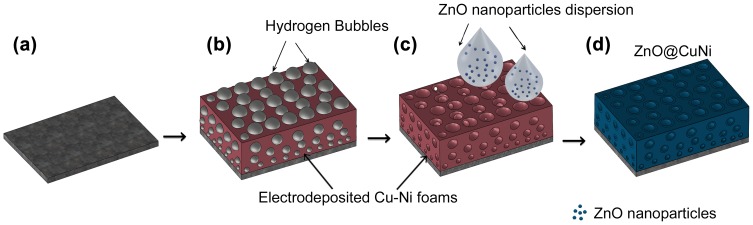
Schematic flow of the synthesis of the ZnO@CuNi hybrid porous layers. From (a) to (b) hydrogen bubble-assisted electrodeposition of porous CuNi MF and from (c) to (d) impregnation of the CuNi scaffold with ZnO NPs suspension.

Two-phase CuNi metallic foams (MF) were successfully obtained by electrodeposition at different current densities on Ti/Si/Au substrates using H_2_ as a dynamic template (Figure 1(a) and 1(b)). All chemicals were of analytical grade and were used as received without further purification. Electrolyte solution was prepared from ultrapure water. Si/Ti (25 nm)/Au (125 nm) substrates (working area of 0.25 cm^2^) were employed as cathodes. Given the low roughness of the Au surface, surface conditioning (e.g. polishing) was not required. All depositions were carried out in a single-compartment, double-jacketed glass electrochemical cell. A platinum sheet served as the counter electrode, and a double junction Ag|AgCl 3 M KCl electrode (E = +0.210 V versus standard hydrogen electrode (SHE)) was utilized as the reference electrode. For CuNi MF electrodeposition, a constant current density of −1 A cm^−2^ and 300 s (Cu_80_Ni_20_) or −3 A cm^−2^ and 100 s (Cu_65_Ni_35_) was applied by using a PGSTAT302 N Autolab potentiostat/ galvanostat (Ecochemie, Utrecht, Netherlands). The deposition charge for the growth of CuNi MFs was kept constant in order to achieve a similar thickness (ca. 60 µm). The electrolyte consisted of CuSO_4_·5H_2_O (0.01 M), NiSO_4_·6H_2_O (0.15 M), H_2_SO_4_ (1 M), CH_3_COOH (0.1 M), HCl (50 mM), and sodium citrate tribasic dehydrate (0.2 M). All deposition processes were carried out at 25°C and under stirring (800 rpm) using a magnetic stirrer bar.

### 2.2. Synthesis of the ZnO nanoparticles

Into a flask containing 20 ml of methanol were added 0.090 g of zinc acetate ([Zn(CH_3_COO)_2_]·2H_2_O Aldrich, 98%) and 25 μl of deionized water. The solution was heated to 60°C under magnetic stirring. Later, a solution of 0.050 g of potassium hydroxide (KOH, Aldrich, 85%) and 20 ml of methanol heated to 60°C was dropwise added to the zinc solution. During this step, ZnO nanoparticles (NPs) were formed by sol-gel (ZnO NPs mean size = 7.3 nm). When the reaction was finished (24 h), the upper fraction of the reaction solution was withdrawn. Next, 40 ml of methanol were added to the solution and stirred for 15 min. Again, the upper fraction of the solution was discarded after 15 min. The methanol washing process was repeated twice. The isolated ZnO NPs were suspended in 40 ml of methanol, leading to a concentration of ca. 0.83 g ZnO l^–1^.

### 2.3. Synthesis of the hybrid ZnO@CuNi porous film

As shown in Figure 1(c) and 1(d), 15 μl of the previously synthesized ZnO NPs suspension were dropwise added onto the surface of the CuNi MF. Next, the impregnated CuNi MF was placed inside a vacuum desiccator (0.5 mbar) for 120 s at room temperature. The impregnation process was consecutively repeated six times to yield ZnO@CuNi porous films with increasingly higher ZnO coverage. Samples were investigated after three and six consecutive impregnations (*middle* and *final* coverage, respectively). Samples were done in triplicate in order to ensure reproducible results.

### 2.4. Morphological and structural characterization

The morphology and structure of the porous films were studied by field emission scanning electron microscopy (FE-SEM), high resolution transmission electron microscopy (HRTEM) and X-ray diffraction (XRD). HRTEM characterization was carried out on a JEOL JEM-2011 microscope operated at 200 kV. For HRTEM observations, a portion of the film was scratched off with a metallic spatula and the powder released was dispersed in ethanol. The dispersion was then sonicated on a SELECTA Ultrasounds 230 V – 40 kHz apparatus (JP Selecta, Barcelona, Spain) for 10 s in order to break up big aggregates. Afterwards, one or two drops of the suspension were placed dropwise onto a holey carbon-coated Cu TEM grid. The chemical composition of the films was determined by energy dispersive X-ray spectroscopy (EDX). The stoichiometry of the Cu-Ni MFs is given in at%. XRD patterns were recorded on a Philips X’Pert diffractometer (Panalytical, Madrid, Spain) using the Cu K_α_ radiation, in the 20–90° 2θ range (0.03° step size, 8 s holding time). A full-pattern fitting procedure (Rietveld method, MAUD software)^33^ was used to extract microstructural parameters from the XRD patterns.

### 2.5. Magnetic characterization

Room temperature hysteresis loops were collected at room temperature using a vibrating sample magnetometer (VSM) from Oxford Instruments (Abingdon), with a maximum applied magnetic field of 0.3 T. The field was applied parallel to the film plane.

### 2.6. Confocal imaging

The photoluminescence (PL) properties of the films were studied by confocal scanning laser microscopy (CSLM). The samples were mounted on Ibidi culture dishes (Ibidi GmbH, Martinsried, Germany). For 3D analysis, the samples were visualized with a TCS-SP5 CSLM microscope (Leica Microsystems CMS GmbH, Mannheim, Germany) using a Plan Apochromat (Leica, Mannhein, Germany) 10× / 0.4 (dry) objective. The ZnO@CuNi nanocomposites were excited with a blue diode laser (405 nm) and the autofluorescence was detected in the 420–580 nm range. Because the CuNi MF matrices are non-fluorescent, they were imaged using reflected light mode from an argon laser (488 nm) and subsequently detected in the 480–495 nm range. Stacks of 50 sections were collected at every 3 μm along the material’s thickness. The three-dimensional images were processed by using Surpass Module in Imaris X64 v. 6.2.0 software (Bitplane; Zürich, Switzerland).

The emission signal of the specimens was excited at 405 nm with a blue diode laser, and its fluorescence intensity was recorded generating a lambda stack (69 images) with emission wavelength ranging from 425 to 750 nm, at 10 nm intervals. A set of 10 regions of interest (ROIs) of 400 μm^2^ each was used to analyze the mean fluorescence intensity (MFI) of the samples in relation to emission wavelength. For each ROI, a graph plotting mean pixel intensity and emission wavelength of the lambda stack was generated.

Finally, photostability experiments were performed to monitor long time-lapse experiments. A defined region of interest (ROI: 620 μm × 620 μm) was photo-bleached at full laser power of 50 mW of blue diode 405 nm (100% power, 100% transmission). Fluorescence signal was detected in the range from 420 nm to 580 nm. Confocal time series were recorded with intervals of one frame / 850 ms for a period of 10 min. Data from all studies were analyzed using the LAS AF software 2.4.1 (Leica Microsystems) obtaining the variation of the MFI (normalized fluorescence values) as a function of irradiation time.

### 2.7. Evaluation of photocatalytic activity

The photocatalytic activity of the uncoated CuNi and hybrid ZnO@CuNi porous layers was evaluated by decolorization of a 5 ppm RhB aqueous solution (Alfa Aesar, Karlsruhe, Germany, 98%, without further treatment). The solutions were prepared by introducing the layers to be tested in 10 ml of the RhB solution. For each experiment, a blank RhB solution was used as a control and reference test. The reaction cells were placed in a SwiftCure IB irradiation cabin (Peschl Ultraviolet GmbH from Mainz, Germany) equipped with a mercury lamp. Cut-off filters were used to limit the wavelength radiation and to avoid direct photolysis of the dye (ISO 10678:2010 standard). The average light intensity used was 220 W and the wavelength ranged from 320 to 500 nm. The photocatalytic experiments were conducted under continuous magnetic stirring at a constant temperature of 29 °C.

After conditioning the suspensions for 60 min in dark to reach adsorption-desorption equilibrium, the light was turned on to initiate the reaction. Experiments were then carried out under ultraviolet-visible (UV-vis) irradiation for an overall time of 180 min. The red color of the solution faded gradually with time due to the decomposition of RhB. Aliquots (4 ml) were withdrawn regularly (at 0, 15, 30, 60, 120, and 180 min) from the reaction. The supernatant solutions were then tested with a UV-vis spectrophotometer (Shimadzu UV-1603, Shimadzu Corporation, Kyoto, Japan) by measuring absorption spectra of RhB (λ = 554 nm) as a function of the irradiation time. Following the measurement, the aliquot was immediately returned to the reaction vessel to continue the reaction. Thus, the final volume marginally decreased (approximately 5%). Photocatalytic activity of the catalysts was calculated as (C/C_o_), where C_o_ is the concentration of the test solution of RhB before irradiation and C is the concentration of RhB after UV-vis irradiation.

## 3. Results and discussion

### 3.1. Morphological and structural characteristics of the porous layers

**Figure 2.**
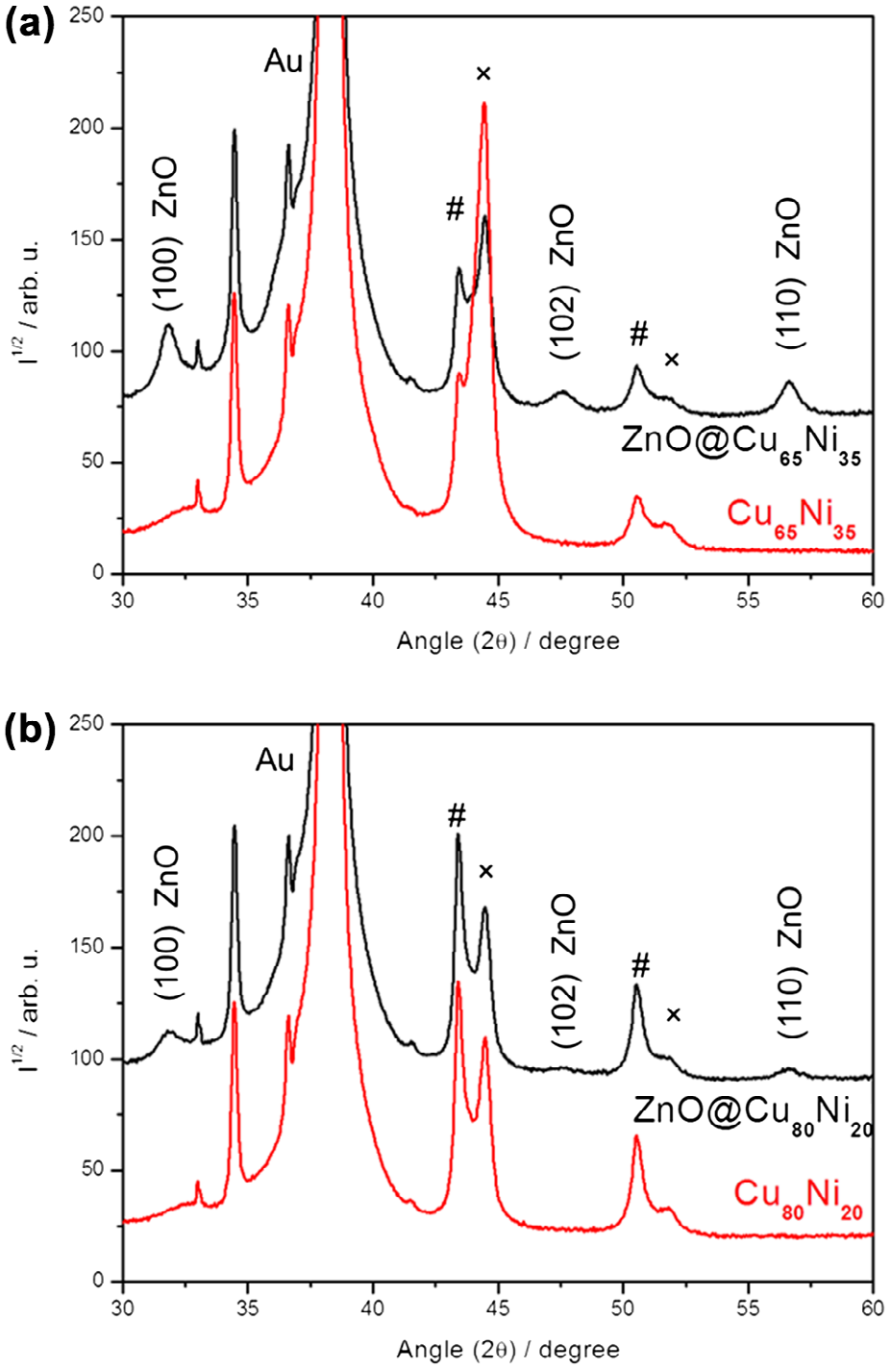
XRD patterns of (a) Cu_65_Ni_35_ (red) and ZnO@Cu_65_Ni_35_ (black) and (b) Cu_80_Ni_20_ (red) and ZnO@Cu_80_Ni_20_ (black). Peaks denoted by # and × belong to Cu-rich and Ni-rich phases, respectively. Two diffraction peaks at 33.06° and 34.36° belong to (100) Si and (100) α-Ti crystalline planes, respectively, of the substrate. Abbreviation: au = arbitrary units.

XRD analyses were carried out to investigate the crystallographic structure of the prepared films. XRD patterns in the 30–60° 2θ range of Cu_65_Ni_35_ (fabricated with j = -3 A cm^−2^) and Cu_80_Ni_20_ (fabricated with j = -1 A cm^−2^) samples before the ZnO deposition are shown in Figure 2. All the samples show four diffraction peaks corresponding to Cu/Ni (111) and Cu/Ni (200) reflections (# and ×, respectively), proving the occurrence of two face-centered cubic (fcc) structures, one enriched in Cu and the other in Ni. The occurrence of two-phase CuNi films was anticipated considering the electrodeposition conditions. Namely, in order to favor intensive hydrogen co-evolution during electrodeposition, a highly acidic electrolyte was employed. In these conditions, citrate has negligible complexing capacity because it exists mostly in its protonated form. As a result, the co-deposition of Cu and Ni cations (and hence, the formation of a solid solution) is severely hindered, which results in heterogeneous nucleation (and hence, the occurrence of phase-separated deposits). From the magnetic viewpoint, formation of phase-separated CuNi films is desirable since room-temperature ferromagnetic properties are then accessible for almost any Ni content. If a single-phase alloy were formed, the ferromagnetic behavior would hold only for Ni percentages above 70 at%. Below this value, alloy layers would be paramagnetic.[34] The average crystallite size of the CuNi parent templates, determined using the MAUD software, ranges between 10 nm and 13 nm for both Cu-rich and Ni-rich phases. By applying the Bragg’s and Vegard’s laws to the (111) reflection, the amount of the Cu- and the Ni-rich phases was estimated to be 85 at% and 15 at%, respectively, for the Cu_80_Ni_20_ film, and 73 at% and 27 at%, respectively, for the Cu_65_Ni_35_ film. The precise stoichiometry of the Cu-rich and Ni-rich phases is given in the Supporting Information (Table S1). The XRD patterns of the hybrid ZnO@CuNi porous layers show, apart from the reflections coming from the metallic Cu-Ni, three additional diffraction peaks at 31.81°, 47.60°, and 56.69°, corresponding to (100), (102) and (110) crystalline planes, respectively, of ZnO in its wurtzite hexagonal phase (JCPDS 36–1451). These peaks are considerably broader, evidencing the nanocrystalline nature of the ZnO coating. Its crystallite size is around 7 nm, as estimated from the Rietveld refinements. Notice that the structural properties of the porous CuNi templates do not become altered after the coating with ZnO, as expected from the room temperature conditions used for ZnO deposition onto CuNi MF.

**Figure 3.**
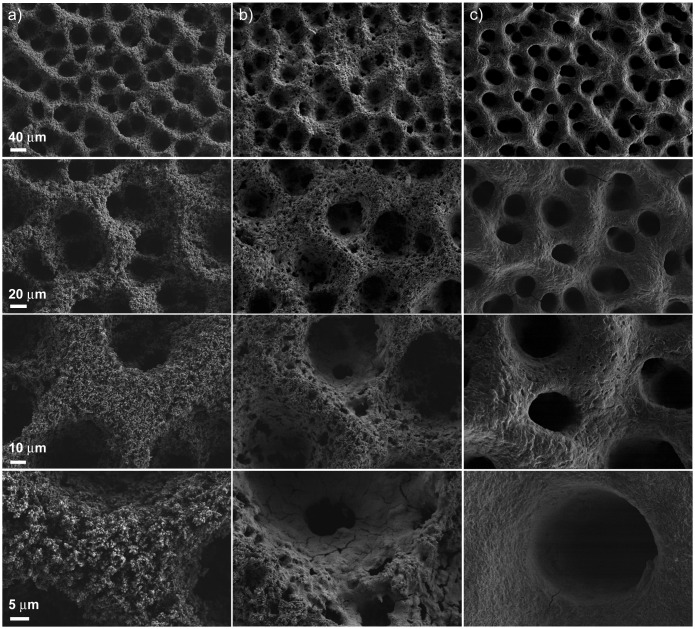
FE-SEM images of (a) the uncoated Cu_80_Ni_20_ MF; and the ZnO-coated Cu_80_Ni_20_ after (b) three consecutive impregnations (*middle* coverage) and (c) six consecutive impregnations (*final* coverage). In each column different magnifications of the three materials are shown.

The evolution of surface morphology from the pristine CuNi MF film upon ZnO impregnation was studied by FE-SEM at different magnifications. As an example, representative FE-SEM images of the uncoated Cu_80_Ni_20_ MF and the corresponding film after being coated with ZnO (three and six impregnation steps or, in other words, *middle* and *final* coverages) are shown in Figure 3. At low magnification, a uniform distribution of 3D interconnected spherical pores of around 50 μm in diameter, was observed over the entire surface of the uncoated MF (Figure 3(a)). In close-up observations, the pore walls (about 20 μm in thickness) were found to be inherently porous (they are built up from numerous nanodendrites, see Figure S1), hence displaying a hierarchical porosity. The size of the 50 μm macropores directly correlates with the size of the H_2_ bubbles. In the present work, acetic acid was added to the bath to prevent from bubble coalescence and, hence, formation of bigger pores. Instead, the porosity of the walls is likely due to the fast deposition rate. CuNi grains cannot stack in a dense manner (as would occur under lower deposition rates) and dendrites are formed. Due to the fractal architecture of such dendrites, numerous ‘nano’ voids are generated during growth.

Upon ZnO impregnation, the nanodendrites become less apparent and finally are no longer visible (Figure 3(b) and 3(c)). As a result, the pore walls show a smoother appearance. Nonetheless, the macro-porosity is preserved along the coating process. These macropores become smaller in size (from about 50 μm in Cu_80_Ni_20_ to about 30 μm in ZnO@Cu_80_Ni_20_) as expected from the increasingly thicker ZnO shell which conformally coats the CuNi network. Representative images of ZnO@Cu_65_Ni_35_ film are shown in Figure S2. In this case, the ZnO shell is less uniform than for ZnO@Cu_80_Ni_20_, which is in part due to the less regular morphology of the CuNi foams featuring larger Ni contents.[35] This is because there is strong dependence of the Ni content on the CuNi morphology. Cu deposition typically promotes the formation of a uniform foam-like architecture. With increasing the Ni content, pore interconnectivity worsens. EDX mapping images prove that Zn is well distributed among the CuNi MF (Figure S3). It is envisaged that the 3D interconnected macroporous structure of the ZnO@CuNi framework could be advantageous for catalytic applications. In particular, an enhanced surface diffusion of the reactant adatoms, ad-ions or molecules through the porous channels and subsequent desorption of the products could positively impact the kinetics of the reaction.

**Figure 4.**
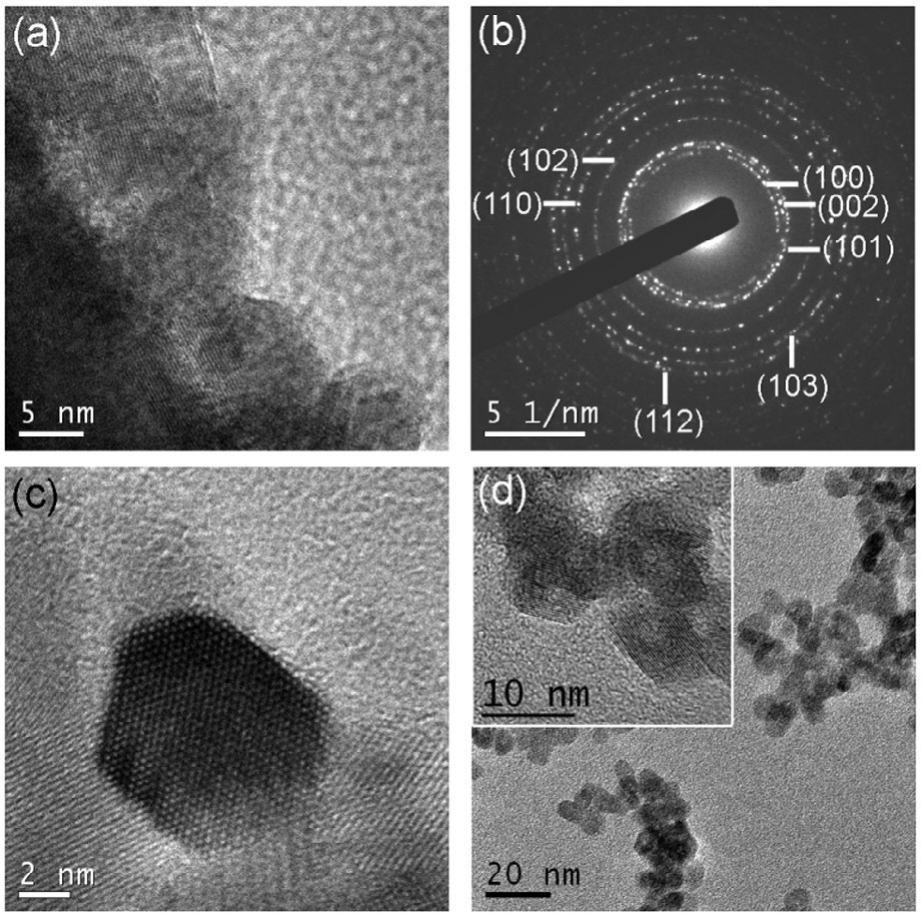
(a) High-resolution TEM image of ZnO@Cu_80_Ni_20_ hybrid porous layer; (b) the SAED pattern of the ZnO NPs stacked on the CuNi layer. (c) High-resolution TEM image of an individual ZnO NP. (d) TEM image of as-prepared ZnO nanoparticles; magnified in the inset.

Figure 4 depicts HR-TEM images of the ZnO@Cu_80_Ni_20_ hybrid porous layer taken at the oxide-metal edge. Figure 4(a) shows that the nanocomposite consists of a rather continuous shell of ZnO NPs well attached to the CuNi MF surface even after sonication of the sample (Figure S4). The selected area electron diffraction (SAED) pattern showed in Figure 4(b) displays spotty rings. Since the spot size was around 200 nm, the beam is actually embracing several small crystals with different orientations. This further confirms the nanocrystalline structure of the ZnO, in agreement with XRD analyses. Furthermore, in a zoomed detail of a single ZnO NP (Figure 4(c)) the lattice planes are sharply defined, thus confirming the crystallinity of the ZnO in the ZnO@Cu_80_Ni_20_ nanocomposite. Interestingly, this synthetic methodology preserves the morphology as well as the crystallinity of the as-prepared ZnO NPs (Figure 4(d)).

### 3.2. Magnetic properties of the porous layers

**Figure 5.**
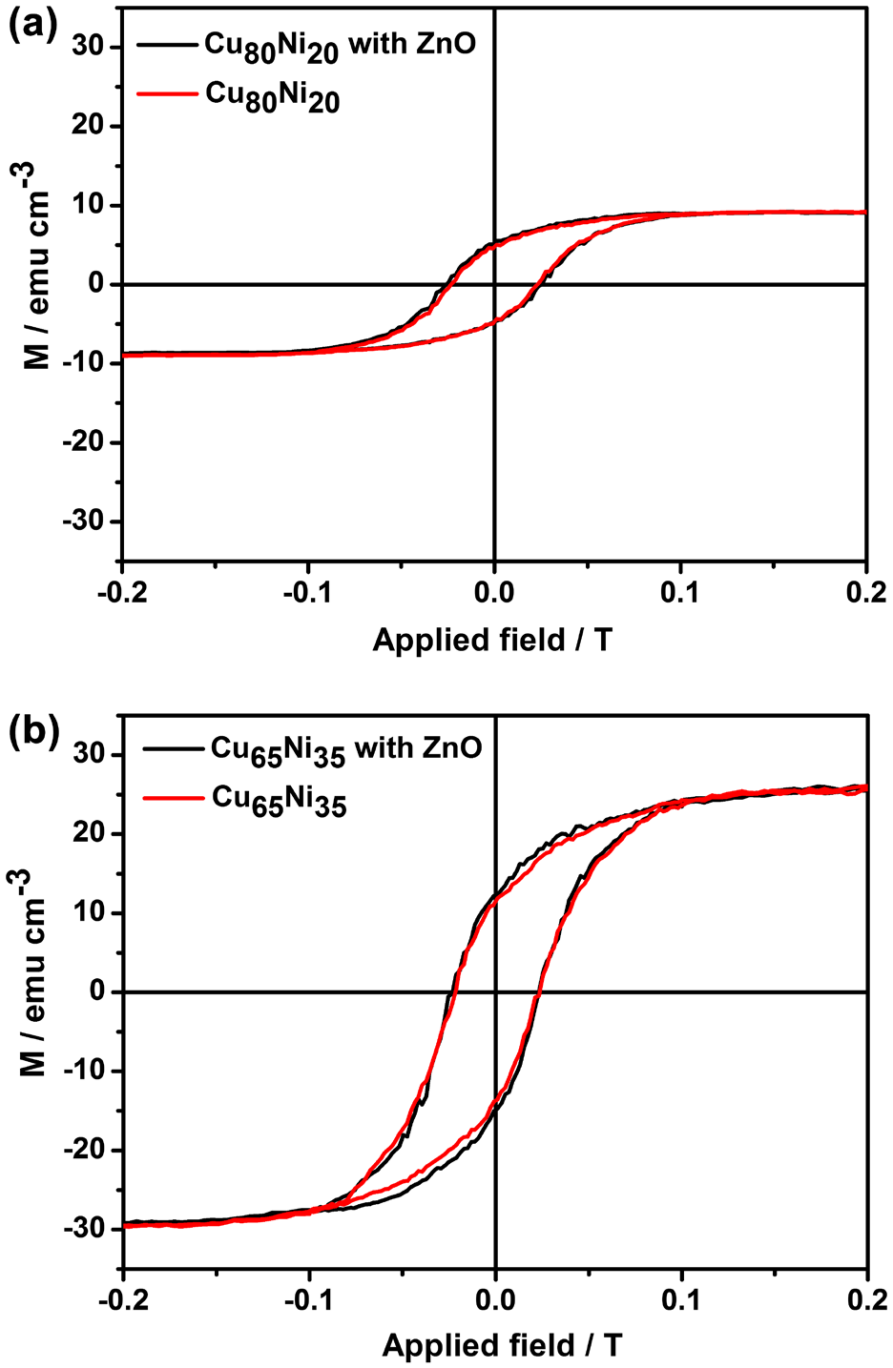
Room temperature hysteresis loops of the (a) Cu_80_Ni_20_ and ZnO@Cu_80_Ni_20_ and (b) Cu_65_Ni_35_ and ZnO@Cu_65_Ni_35_ samples.

Well-defined hysteresis loops corresponding to the Cu_80_Ni_20_ MFs as well as ZnO@Cu_80_Ni_20_ are shown in Figure 5(a). It is known that CuNi single-phase alloys are ferromagnetic at room temperature for Ni contents above 70% at.[34, 36] Hence, the occurrence of ferromagnetism in spite of the low Ni content is a further indication that the CuNi MF are not single phase but, instead, Ni-rich clusters (ferromagnetic) co-exist together with a non-ferromagnetic Cu-rich matrix, in agreement with XRD results (Figure 2). To further explore the influence of the Ni content on the magnetic response of the layers, uncoated and ZnO-coated Cu_65_Ni_35_ films were also investigated. The Cu_65_Ni_35_ MF and its homologous ZnO@Cu_65_Ni_35_ porous film (Figure 5(b)) exhibit higher saturation magnetization (M_S_) in comparison to Cu_80_Ni_20_ based films due to the higher Ni content. The hysteresis loops also reveal that the coercivity, H_C_, tends to slightly decrease with the increase of the Ni content in the nanocomposites, in agreement with our previous work on uncoated CuNi porous films.[35] Remarkably, for both compositions the presence of the ZnO shell does not alter the magnetic response of the CuNi matrix as M_S_ remains the same before and after the ZnO coating. This indicates that the metallic character of the pristine CuNi MF is maintained during ZnO impregnation (i.e. no oxidation occurs). Hence, this synthetic approach can be of high interest in applications where it is necessary to retain the initial magnetic properties of the MF. Since the ZnO coating over the Cu_65_Ni_35_ MF was defective (Figure S1) or, at least, less conformal than over the Cu_80_Ni_20_ MF, the latter was selected for the subsequent analysis of the optical and catalytic properties.

### 3.3. Optical properties of the hybrid layers

**Figure 6.**
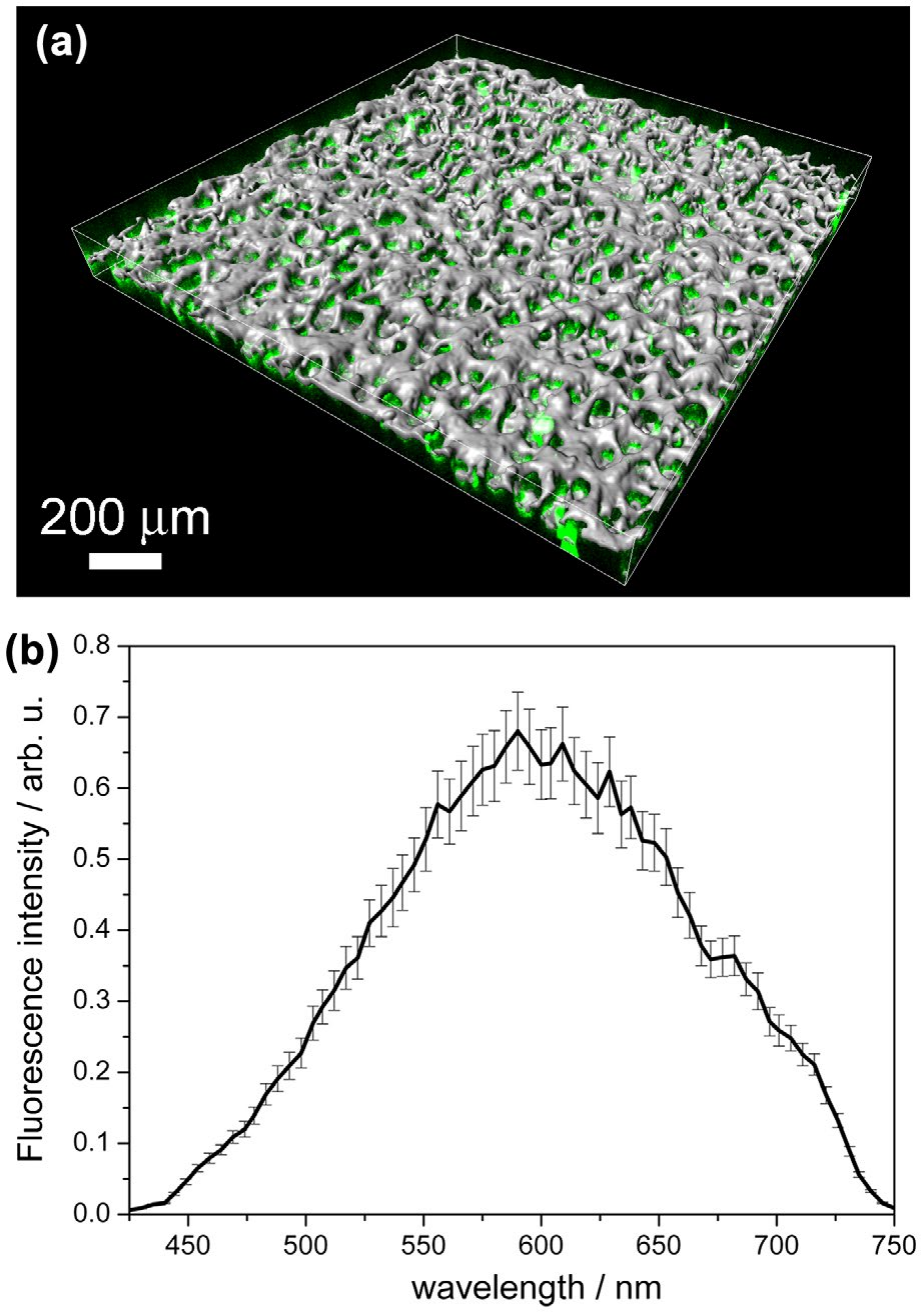
(a) 3D projection images of the hybrid ZnO@Cu_80_Ni_20_ nanocomposite isosurface view by Imaris v.6.2.0 software. The 3D representation was obtained from a 50-section stack in steps of 3 μm. CuNi MF shows as gray color (reflection mode) whereas the ZnO component shows as green color (fluorescence). (b) Fluorescence spectrum obtained with the Lambda scan module of CSLM at a 5 nm resolution. Abbreviation: au = arbitrary units.

**Figure 7.**
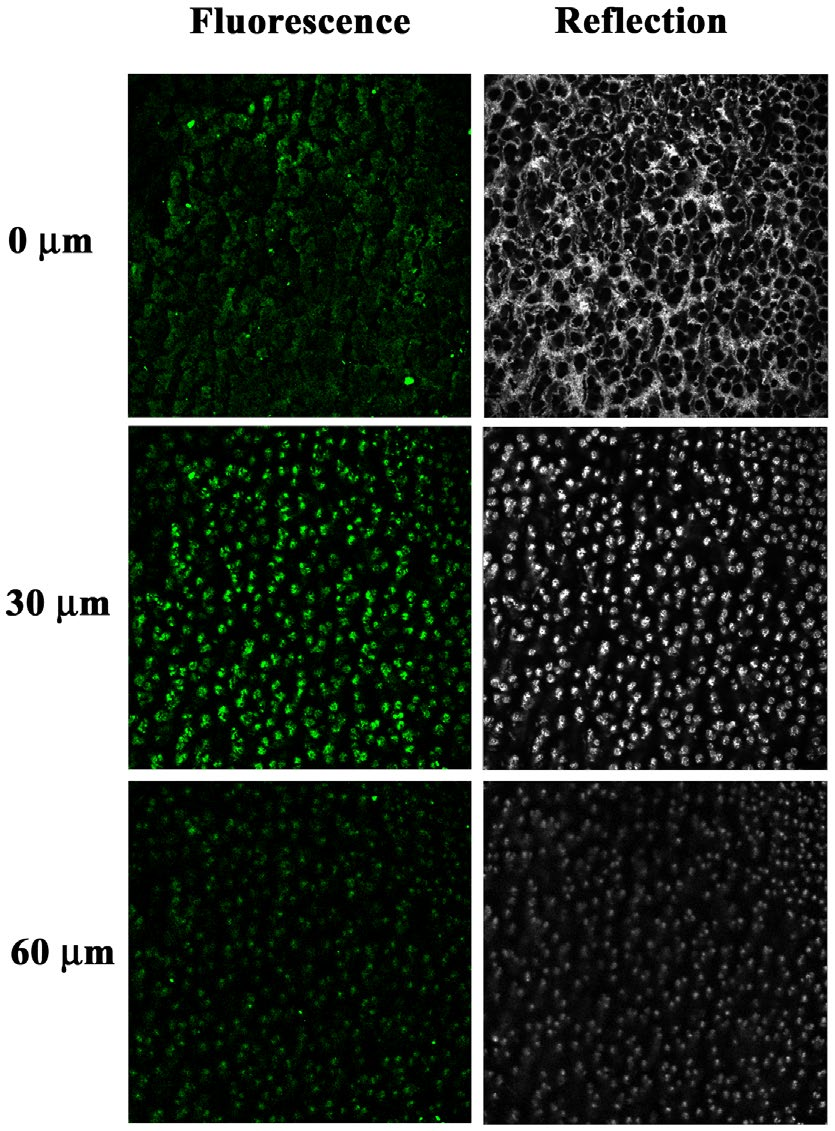
Fluorescence and reflection confocal micrographs (sample area: 621 μm x 621 μm) of the ZnO@Cu_80_Ni_20_ hybrid porous layer taken at different sections from the nanocomposite surface up to a total depth of 60 μm. Fluorescence microscopy was employed to visualize ZnO NP (in green). Reflection confocal microscopy was used to visualize the CuNi matrix (in gray).

3D image reconstruction and confocal methodologies offer great advantages for studying complex nanocomposites without mechanical distortions, providing valuable 3D information. Figure 6(a) shows the isosurface module of Imaris 3D photoluminescence (3D-PL, green) and structural (reflection mode, gray) image of the porous ZnO@Cu_80_Ni_20_ porous nanocomposite film. The full dataset was surveyed to determine the range of intensities (gray values) within the volume (0–255; black–white). An isovalue (intensity) was chosen and the software constructed a map of all the chosen values in the volume. After segmentation, isosurfacing produces simplified hollow objects (or 3D surfaces), which are accurate simplified representations of the original shape of the segmented structure. This surface model was extremely helpful to study the 3D shape and spatial relationship with different layers of the hybrid nanocomposite. Notice that the green (arising from the ZnO) and the gray (arising from the CuNi) colors indicate a homogeneous distribution of ZnO within the CuNi matrix. Particularly, the PL response (Figure 6(b)) consists of broad-band emission which covers the entire visible range (in the wavelength interval 425–750 nm) and has a maximum of around 595 nm. In general, a broad green fluorescence in ZnO is attributed to oxygen defects.[37] Moreover, the PL signal is recovered across the entire thickness of the porous layer as can be observed in Figure 7. The intrinsic fluorescence follows the distribution of the reflection signal although it is more intense at 30 μm depth. A decay of the signal is observed at a higher depth. Nevertheless, this indicates that the ZnO is not only present at the outermost CuNi surface but it rather penetrates through the entire CuNi network and reaches the Au surface. Therefore, the obtained materials can be truly regarded as 3D porous composites. Interestingly, contrary to fluorescent dots, where a pronounced decrease in the PL intensity is observed during illumination with UV or visible light (photobleaching),[38] here only a decrease of 15% of PL intensity was observed after irradiation during 10 min (Figure S5). Due to this stability in the PL signal and the relatively large width of the observed PL peak, these novel ZnO@CuNi porous films could be appealing for optoelectronic devices.

### 3.4. Photocatalytic degradation of RhB dye

The photocatalytic performance of the ZnO@Cu_80_Ni_20_ porous film was evaluated by assessing the degradation rate of RhB under UV-vis irradiation (320 < *λ* < 500 nm) after the adsorption–desorption equilibrium was reached.

**Figure 8.**
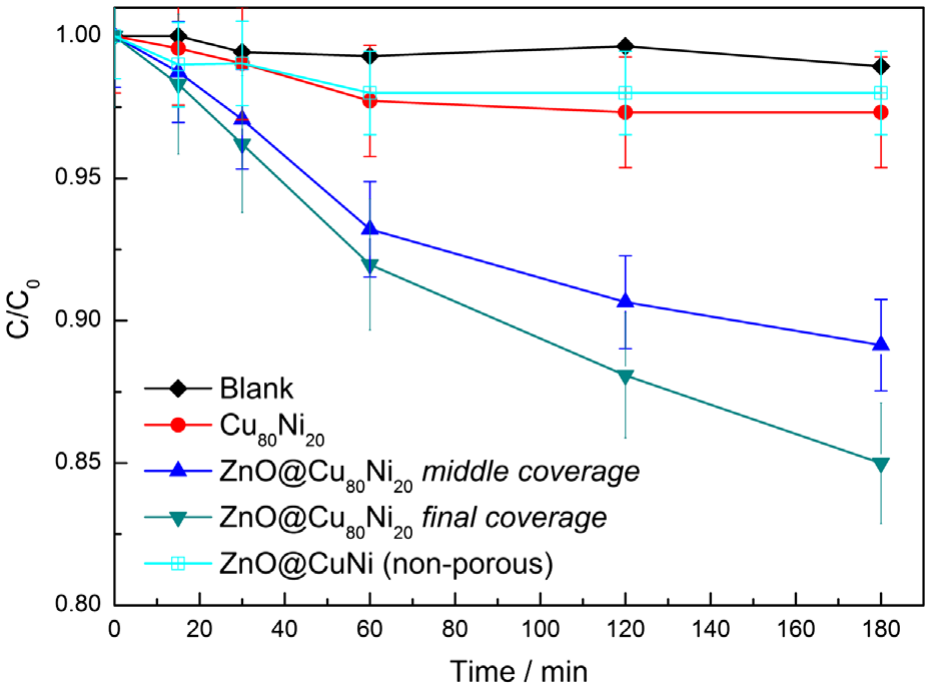
C/C_0_ plot of the degradation of RhB in the blank, in the presence of Cu_80_Ni_20_ MF, and in the presence of ZnO@Cu_80_Ni_20_ photocatalysts with *middle* and *final* coverages. The RhB degradation profile for ZnO-coated non-porous Cu-rich flat layer is also shown for comparison.

As can be observed in Figure 8, the control test without catalysts (blank) under UV-vis light irradiation showed that the photolysis of RhB is negligible. On the other hand, for the uncoated CuNi MF the concentration of RhB decreased very slightly: only 2% of the original organic matter was degraded after 3 h of exposure time. Regarding the ZnO-coated CuNi, it can be observed that the relative concentration (C/C_0_) of RhB dye markedly decreased with the increase of exposition time. The final C/C_0_ value was a function of the ZnO amount. The RhB concentration in solution decreases by 10% and 15% after 3 h when ZnO@Cu_80_Ni_20_ with *middle* and *final* coverages, respectively, were used. As expected, the photoactivity of hybrid porous ZnO@CuNi thin films is higher than that of bare CuNi MF, and it increases with ZnO content. Furthermore, the activity of a ZnO-coated non-porous electroplated CuNi flat dense film is much lower. This puts forward the importance of a large specific surface area for efficient dye removal and suggests that the macropore sidewalls do contribute to the photocatalytic activity. According to the literature,[39] RhB dye degradation mechanism may proceed according to (1–5):

(1) $$ZnO+h\nu \to e_{cb}^{-}+h_{vb\;}^{+}$$

(2) $$O_{2}+e^{-}\to {\;O}_{2}^{\cdot -}+H^{+}\to {HOO}^{\cdot }$$

(3) $$e^{-}+{HOO}^{\cdot }+{\;H}^{+}\to {\;H}_{2}O_{2}$$

(4) $$H_{2}O_{2}+e^{-}\to {\;OH}^{\cdot }+{\;OH}^{-}$$

(5) $$\text{RhB +}\;{\;O}_{2}/O_{2}^{\cdot -}/{HOO}^{\cdot }/{OH}^{-}\;\to \;\;\text{mineralized product}$$

Upon illumination, the generation of electron–hole (e_cb_^–^ and h_vb_^+^) pairs between conduction (CB) and valence bands (VB) of ZnO NPs occurs (Equation (1)). The generated e^–^ are then scavenged by the dissolved O_2_ to generate superoxide anion $O_{2}^{\cdot -}$ (Equation (2)), which, in turn, generate H_2_O_2,_ OH ions and radicals (Equations (3) and (4)). All the generated reactive species are able to degrade RhB and mineralize it (Equation (5)).

Previous conditioning under dark conditions for 60 min in the presence of the ZnO@Cu_80_Ni_20_ did not bring any change in the RhB concentration, indicating that UV-vis irradiation is indispensable for dye degradation. Upon several consecutive degradation tests under irradiation conditions, a decrease of the photocatalytic efficiency was observed (Figure S6), likely due to gradual deactivation of ZnO caused by the adsorption of species present in solution.[40] No apparent detachment of the ZnO NPs from the CuNi MF was observed though. The solution remained fully transparent (eventual turbidity arising from detached ZnO NPs was not noticed) and SEM images of the CuNi MF after the photocatalytic tests did not disclose appreciable changes in morphology. Compared to the literature dealing with ZnO-based materials, the photocatalytic activity of the here-developed hybrid composites is not particularly high. Different strategies could help improve the photocatalytic performance: through doping of ZnO with suitable cations/anions or by further increasing the specific surface area of the CuNi MF (e.g. via the creation of homogeneously distributed nanopores) and, hence, of the ZnO coating. Moreover, diffusion of the RhB dye through the 3D macroporous network is perhaps not fully efficient. Indeed, taking into account the mean pore diameter (several micrometers), it is expected that a non-negligible fraction of the inner part of the film contributes to the photocatalytic activity. If the pores were much narrower, diffusion of RhB through the pores would have been probably more challenging. Nevertheless, detailed studies are required to evaluate the efficiency of dye diffusion within the 3D network. Although the photocatalytic tests with ZnO-coated non-porous CuNi films do indicate that porosity is definitely beneficial, further work should be done in order to take full advantage of the macroporous architecture of the material. This also includes optimizing the way the material is irradiated so that the inner ZnO shells fully contribute to the catalytic response.

## 4. Conclusions

In summary, a fast and inexpensive synthetic approach to obtain hybrid porous ZnO@CuNi films has been presented. First, two-phase CuNi porous magnetic films were grown by means of electrodeposition. Then, a suspension containing ZnO nanoparticles was dropwise added onto the CuNi framework, coating not only its outermost external surface but also the inner pores, as revealed by CLSM imaging, hence rendering truly 3D porous ZnO@CuNi hybrid films. This strategy overcomes the problems associated with high temperature post-synthesis heat treatments that can be deleterious from both morphological and compositional viewpoints. Indeed, thanks to the lack of annealing treatments, the inherent macro-porosity of the initial CuNi MFs as well as their ferromagnetic properties are preserved after coating with ZnO. The obtained ZnO@CuNi hybrid porous layers show some activity for the aqueous degradation of Rhodamine B under UV-vis light irradiation. When supported onto substrates, these materials could be handled as classical heterogeneous catalysts. Compared to ZnO-based powders, tedious recycling steps such as filtration and centrifugation are not required. If released from the substrate, the composite films could eventually be wirelessly manipulated using external magnetic fields, whilst being both photoluminescent and photocatalytic upon irradiation under UV-vis light. This would be possible due to the combination of the various properties of the different phases comprising the hybrid composites. The reported synthetic strategy could also be extrapolated to other types of materials, paving the way for the preparation of hybrid materials in general, suitable for multifunctional solid-state devices.

## Disclosure statement

No potential conflict of interest was reported by the authors.

## Supplementary Material

Supporting Information
