# Supplementary material for: Room-temperature synthesis of three-dimensional porous ZnO@CuNi hybrid magnetic layers with photoluminescent and photocatalytic properties
Source: Sci Technol Adv Mater. 2016 Apr 14;17(1):177–87. doi: 10.1080/14686996.2016.1165583 (PMC5101997; doi:10.1080/14686996.2016.1165583)
Supplement: Supporting Information [file tsta_a_1165583_sm6640.docx]

Supporting Information

**Room Temperature Synthesis of Three-Dimensional Porous ZnO@CuNi Hybrid Magnetic Layers with Photoluminescent and Photocatalytic Properties**

Miguel Guerrero, Jin Zhang, Ainhoa Altube, Eva García-Lecina, Mònica Roldán, Maria Dolors Baró, Eva Pellicer and Jordi Sort

**Figure S1.** SEM images of the Cu_80_Ni_20_ walls showing the dendritic-like morphology.

**
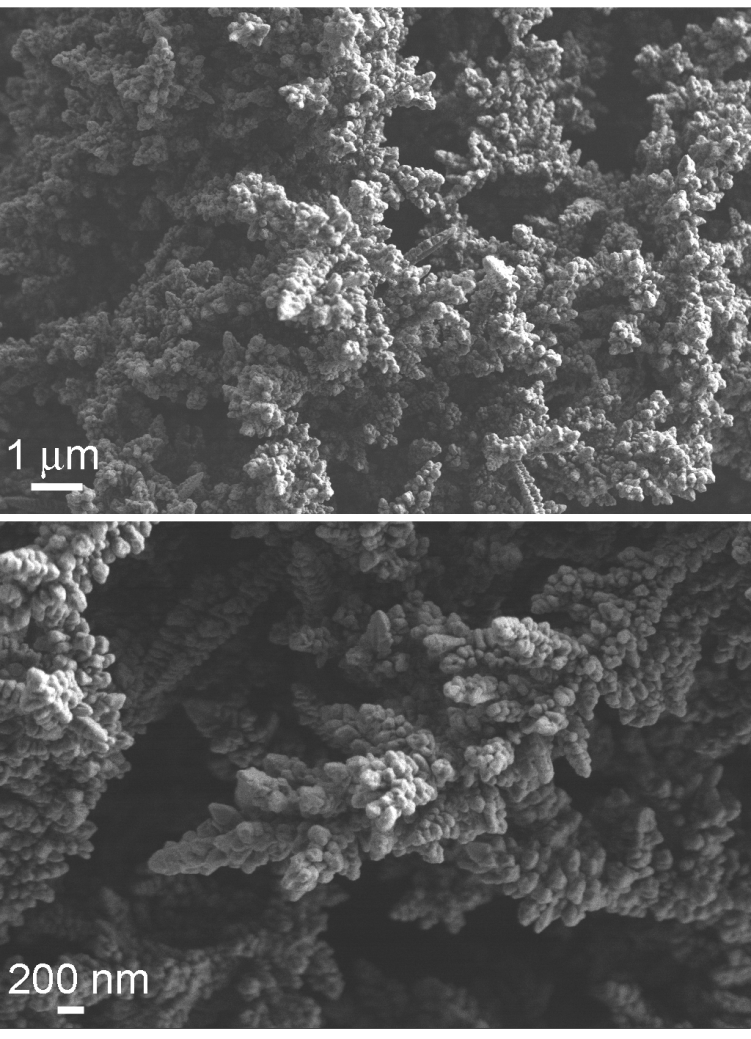
**

**Figure S2.** FE-SEM images at different magnifications of the ZnO-coated Cu_65_Ni_35_ porous layer (*final* coverage).

**
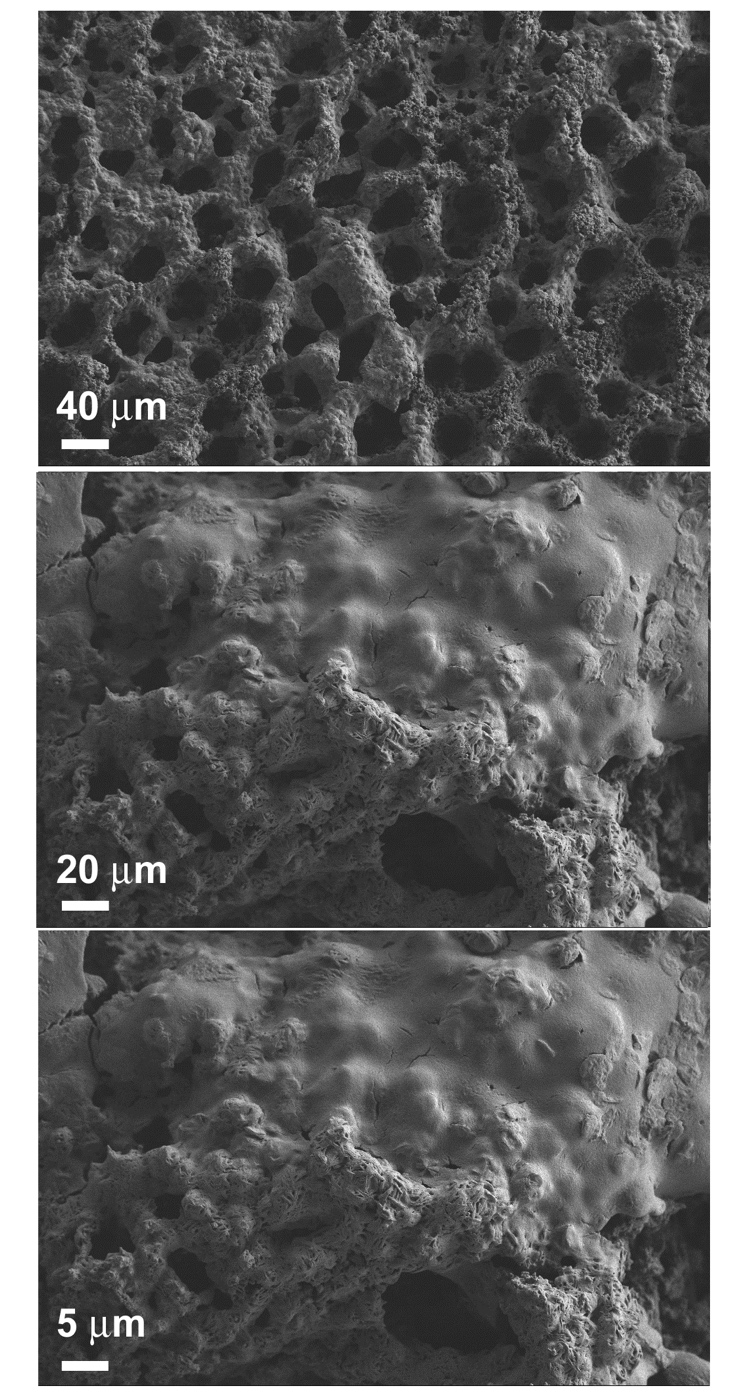
**

**Figure S3.** EDX mapping images of the middle coverage ZnO@Cu_80_Ni_20_ porous nanocomposite film. (a) SEM image, (b) Zn, (c) Cu and (d) Ni element distribution.

**
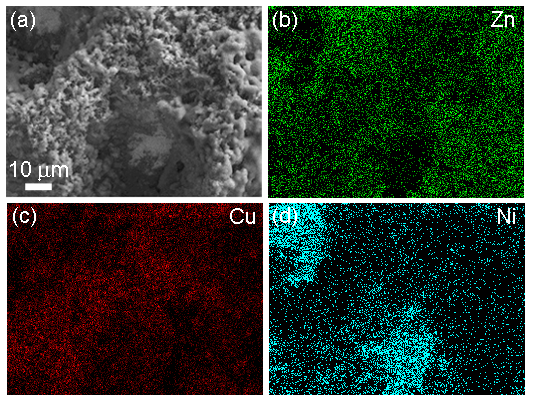
**

**Figure S4.** TEM images of the Cu_80_Ni_20_/ZnO interface, showing the ZnO NPs well stacked to the CuNi layer.

**
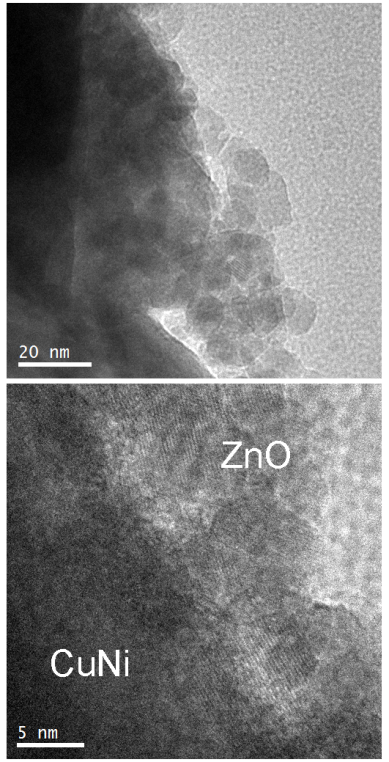
**

**Figure S5.** Photostability of the ZnO@Cu_80_Ni_20_ porous nanocomposite film upon irradiation at its excitation wavelength (405 nm).

**
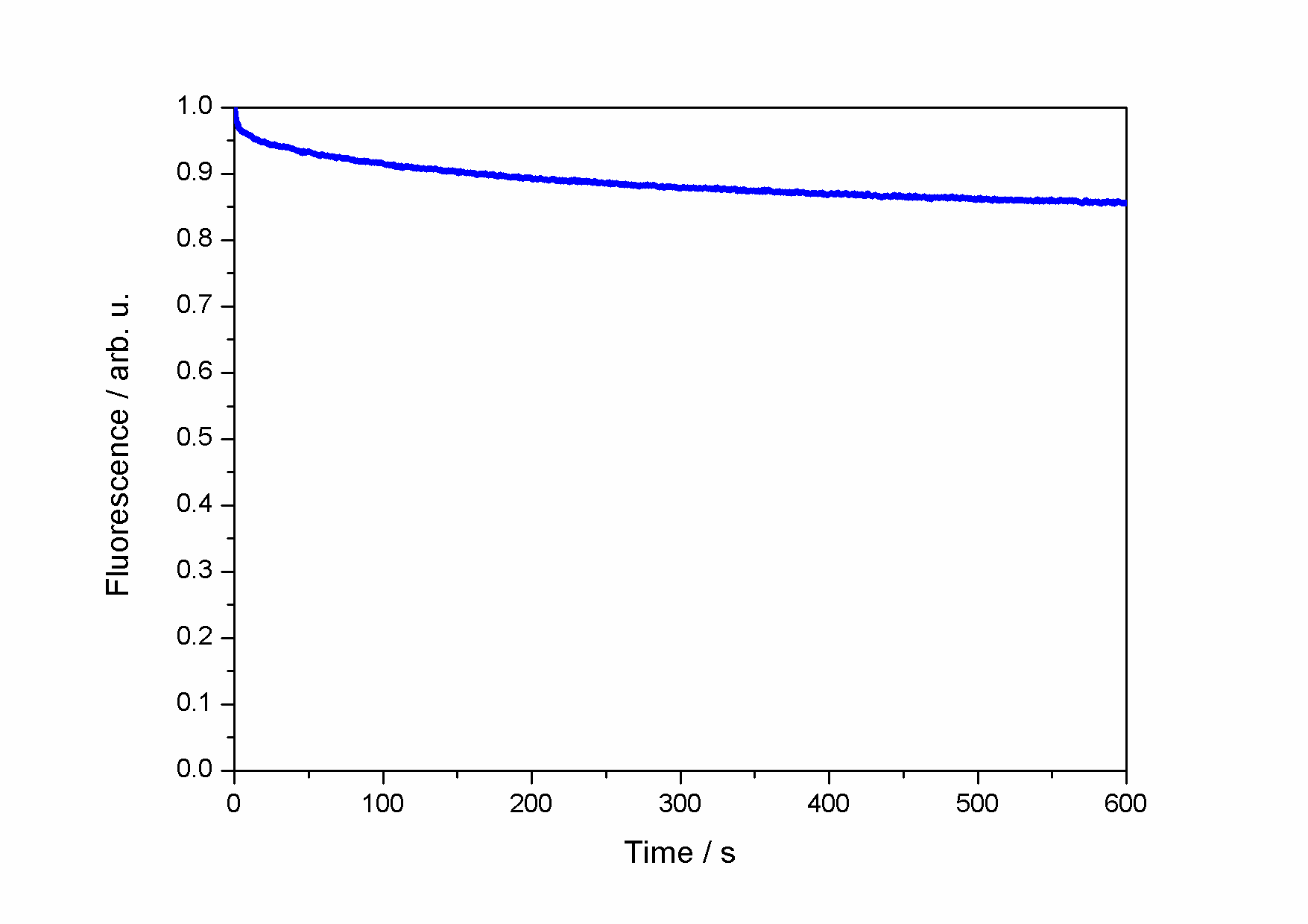
**

**Figure S6.** C/C_0_ plot of the degradation of RhB in the blank, in the presence of Cu_80_Ni_20_ MF, and in the presence of ZnO@Cu_80_Ni_20_ photocatalysts with middle and final coverages at the 5^th^ cycle.





**Table S1**. Composition of the Ni-rich and Cu-rich phases present in the two investigated porous films, as determined from XRD analyses (combining the Bragg’s law with the Vegard’s law).

| Cu-Ni layer | Cu-rich phase | Ni-rich phase |
| --- | --- | --- |
| Cu_80_Ni_20_ | 2θ = 43.39 (deg)  *d* = 2.0839 (Å)  *a* = 3.6094 (Å)  at% Cu = 93.92  at% Ni = 6.08 | 2θ = 44.48 (deg)  *d* = 2.0352 (Å)  *a* = 3.5251 (Å) at% Cu = 1.19  at% Ni = 98.81 |
| Cu_65_Ni_35_ | 2θ = 43.47 (deg)  *d* = 2.0802 (Å)  *a* = 3.6030 (Å)  at% Cu = 86.91  at% Ni = 13.09 | 2θ = 44.43 (deg)  *d* = 2.0374 (Å)  *a* = 3.5289 (Å) at% Cu = 5.43  at% Ni = 94.57 |
